# Observational study of the effects of traumatic injury, haemorrhagic shock and resuscitation on the microcirculation: a protocol for the MICROSHOCK study

**DOI:** 10.1136/bmjopen-2015-010893

**Published:** 2016-03-04

**Authors:** Sam Hutchings, David N Naumann, Tim Harris, Julia Wendon, Mark J Midwinter

**Affiliations:** 1 Kings College Hospital, Denmark Hill, London, UK; 2 Kings College London, London, UK; 3 Royal Centre for Defence Medicine, Queen Elizabeth Hospital, Birmingham, UK; 4 University of Birmingham, Birmingham, UK; 5 University Hospitals Birmingham NHS Foundation Trust, Queen Elizabeth Hospital, Birmingham, UK; 6 Barts Health NHS Trust and Queen Mary University of London, London, UK

**Keywords:** TRAUMA MANAGEMENT, INTENSIVE & CRITICAL CARE, ACCIDENT & EMERGENCY MEDICINE

## Abstract

**Introduction:**

The microcirculation is the physiological site of oxygen and substrate exchange. Its effectiveness during circulatory shock is vital for the perfusion of tissues, and has a bearing on subsequent organ function and prognosis. Microcirculatory dysfunction following traumatic haemorrhagic shock (THS) has been understudied compared with other pathologies such as sepsis. The aim of the MICROSHOCK study is to investigate changes seen in the microcirculation of patients following THS, and to assess its response to resuscitation. A greater understanding of the behaviour and mechanisms of microcirculatory dysfunction in this context may direct future avenues of goal-directed resuscitation for these patients.

**Methods and analysis:**

This multicentre prospective longitudinal observational study includes patients who present as an emergency with THS. Microcirculatory parameters are recorded using sublingual incident dark field microscopy alongside measurements of global flow (oesophageal Doppler and transthoracic echocardiography). Patients are enrolled into the study as soon as feasible after they arrive in hospital, and then at subsequent daily time points. Blood samples are taken for investigation into the mechanisms of microcirculatory dysfunction. Sequential Organ Failure Assessment scores will be analysed with microcirculatory parameters to determine whether they correlate with greater fidelity than more conventional, global circulatory parameters.

**Ethics and dissemination:**

Research Ethics Committee approval has been granted for this study (Reference: 14/YH/0078). Owing to the nature of THS, capacity for informed consent will be absent on patient enrolment. This will be addressed according to the Mental Health Capacity Act 2005. The physician in charge of the patient's care (nominated consultee) may consent on behalf of the patient. Consent will also be sought from a personal consultee (close relative or friend). After capacity is regained, the participant will be asked for their consent. Results will be submitted for publication in peer-reviewed journal format and presented at relevant academic meetings.

**Trial registration number:**

NCT02111109; Pre-results.

**Strengths and limitations of this study**

- This study's main strength is that it will be the largest clinical investigation into the microcirculatory response to traumatic injury and haemorrhagic shock to date.
- The MICROSHOCK study will recruit from major trauma centres in London and Birmingham, the two largest cities in the UK.
- The study is limited by its observational nature. However, the key research question ‘Does the microcirculatory behaviour predict outcome better than global haemodynamic parameters?’ can be addressed using observational data.
- A further limitation is the heterogeneous nature of injury patterns in the UK trauma patient population. Such differences will be addressed during data analysis, and reported during the dissemination of the results. These differences will be much greater than those in animal studies. However, a clinical study allows us to translate what has been learnt from animal research into useful and clinically relevant knowledge.

## Background

Massive haemorrhage and associated shock is a leading cause of preventable death among casualties with severe traumatic injury, accounting for around 40–50% of all deaths.^1^ ^2^ Despite significant improvements in treatment and mortality within this patient group, systemic inflammatory response, coagulopathy and subsequent multiple organ failure remain common.^3^ These issues can lead to prolonged periods of morbidity, critical care dependency and often death. Therapeutic interventions that attenuate these responses may lead to significant benefit for patients who have suffered traumatic haemorrhagic shock (THS).^3^

The microcirculation is of particular clinical importance during circulatory shock since it is this network of capillaries and other small vessels that perform the essential functions of delivering oxygen and substrates to cells. Traditionally, resuscitative strategies are guided by measurement of global haemodynamic parameters such as blood pressure and cardiac output. However, there may be a poor correlation between such global parameters and the appearance of the microcirculation.^4^ ^5^ Microcirculatory parameters may also predict outcome better than more conventional global circulatory measurements in sepsis.^6^ Observational data suggest patients who can improve the state of the microcirculation in response to resuscitative fluid therapy have a better outcome than those who cannot.^7^ ^8^

Incident dark field (IDF) video microscopy is a method of assessing the microcirculation that has been developed relatively recently for clinical and research.^9^ A light source is applied to the tissue, which illuminates the deep tissues within the target field. The reflected light from the deep tissues is captured and magnified before being received on a viewer. The selective wavelength of transmitted light is completely absorbed by both oxygenated and deoxygenated haemoglobin, and blood vessels therefore appear black. The technique is limited to organs that have a thin epithelial covering and that are accessible to a handheld probe, which in clinical practice has usually been the sublingual microcirculation. The device is small, portable and the technique non-invasive. Obtaining images with the device requires between 2 and 5 min and involves the use of a small, non-traumatic sublingual probe. The images produced by IDF microscopy require offline analysis and the ascription of objective values before they can be used. De Backer *et al*^10^ have standardised this approach.

Most clinical studies that examine the microcirculation have focused on sepsis,^7^ ^11^ ^12^ whereas studies relating to haemorrhagic shock are mostly limited to animal experiments, and have usually looked at controlled haemorrhagic shock rather than a complex traumatic injury model.^13–15^ One clinical study has examined the microcirculation after THS once the patients had been admitted to the intensive care unit (ICU), but did not gather data during the early phases of resuscitation.^16^ Although they share similarities, septic and haemorrhagic shock must be examined separately in a clinical context, so that their respective differences can be delineated, and unanswered questions can be addressed.

### Primary objective

The primary objective of the MICROSHOCK study is to examine the microcirculatory function in patients following THS, and to determine whether these parameters are superior to global haemodynamic parameters in the prediction of clinical outcomes.

## Methods

### Study design

This is a multicentre prospective longitudinal observational study that involves serial assessments of the sublingual microcirculation alongside measurements of global flow, volume status and blood pressure, determined from existing monitoring devices and using focused transthoracic echocardiography. In conjunction, data will be gathered relating to the patients physiology and resuscitation.

### Patient screening

All patients with a history of traumatic injury presenting to the emergency department (ED) at the study site hospitals will be screened by research staff. These sites are all UK Major Trauma Centres, and include (1) Kings College Hospital, London, UK; (2) The Royal London Hospital, London, UK; and (3) Queen Elizabeth Hospital Birmingham, Birmingham, UK. Patient details will be recorded in a screening log.

### Number of patients

A single pilot study demonstrated feasibility in the examination of the microcirculation following THS, and reported data for 18 patients.^16^ The current study will be the first to examine the microcirculation of patients with THS as soon as they have arrived in the ED. The MICROSHOCK study will enrol 60 patients in order to further investigate the microcirculation following THS, and form hypotheses that might direct future goal-directed interventions.

### Inclusion criteria

Adult patients with evidence of haemorrhagic shock exhibiting all of the following features:

1. Mechanism of injury consistent with blood loss;
2. Intubated and ventilated;
3. Serum lactate concentration >2 mmol/L recorded at any stage prior to admission to the ICU;
4. Have received any blood products (eg, packed red blood cells (PRBC), fresh frozen plasma (FFP), cryoprecipitate, platelets) during the initial period of resuscitation, prior to admission to ICU, or are predicted to receive blood products during this timeframe in the opinion of the trauma team leader.

### Exclusion criteria

Patients with facial injuries, where access to the sublingual area would be problematic will be excluded. Patients with injuries deemed unsurvivable in whom the focus of care is palliation rather than active treatment are also ineligible for inclusion.

### Time points and techniques for data collection

**Figure 1:**
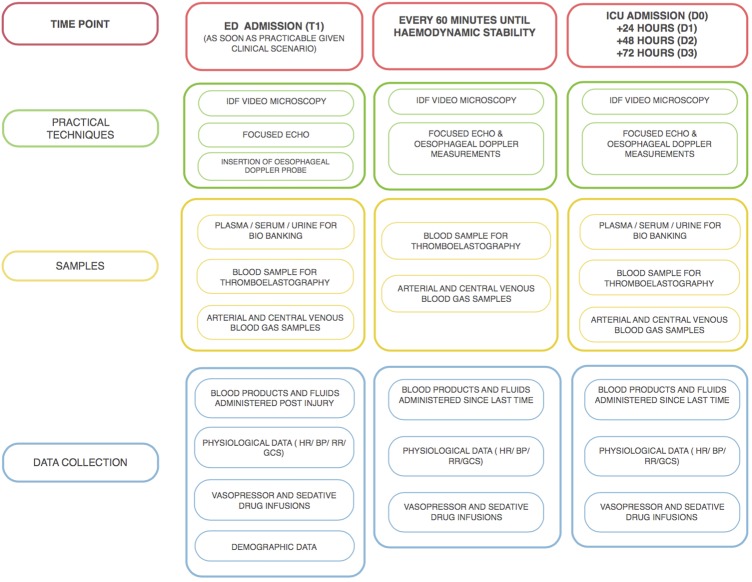
Sampling and techniques. BP, blood pressure; ED, emergency department; GCS, Glasgow Comas Scale; ICU, intensive care unit; IDF, incident dark field; HR, heart rate; RR, respiratory rate.

Figure 1 outlines the techniques that will be utilised, samples taken and data collected at each time point. There are five main time points:

1. As soon as feasible after arrival in hospital (T1), and up to every 60 min until haemodynamically stable;
2. On admission to ICU (D0);
3. 24 h after D0;
4. 48 h after D0;
5. 72 h after D0.

Where feasible, and appropriate, further readings will be taken between the first and second time points. Data collection is stopped once the patient has been extubated.

### Demographic and resuscitation data

The following baseline (hospital admission) demographic and physiological data will be collected from study participants and recorded in an electronic database on a secure encrypted computer used only for research purposes. All data in this log will be anonymised and assigned a sequential study number.

- Demographic details (age, gender, ethnicity);
- Mechanism of injury;
- Injury Severity Score (ISS);
- Admission systolic blood pressure (SBP), respiratory rate and Glasgow Comas Scale (GCS; to calculate Trauma and Injury Severity (TRISS) score);
- Administered prestudy blood products and intravenous fluids;
- Timings from injury to start of prehospital resuscitation, ED resuscitation and operating theatre-based resuscitation;
- Nature of initial surgical procedures and treatments.

### Acquisition of IDF images

The investigator will obtain video clips of the sublingual microcirculation using an IDF videomicroscope (Cytocam, Braedius Medical B.V., Huizen, The Netherlands). In accordance with international consensus criteria for assessing the microcirculation, enough video will be recorded to enable at least three (and preferably five) ×10 s video clips to be obtained at each time point. Because the microcirculation can demonstrate considerable heterogeneity, particularly in shock states, images will be acquired from different sites within the sublingual area. When acquiring images, investigators should actively seek to minimise pressure artefact by observing flow in medium and large vessels. The image should be stabilised with minimal movement artefact. Light intensity and focus should be optimised. Individual video sequences will be exported from the image acquisition software as DV-AVI files.

### Acquisition of transthoracic echo images

Subcostal and apical five-chamber windows will be obtained. The inferior vena cava will be visualised in M mode, and the minimum and maximum diameters across the respiratory cycle recorded. The left ventricular outflow tract (LVOT) will be visualised and a pulsed wave Doppler signal acquired. The LVOT velocity time integer (VTi) and the degree of variation across the respiratory cycle will both be recorded.

### Oesophageal Doppler

An oral oesophageal Doppler probe (Deltex Medical, Chichester, UK) will be inserted at the same time as acquisition of the first IDF images. The probe will remain in situ continuously for 72 h or until the patient is ready for extubation. The following data will be recorded: corrected flow time, stroke volume, cardiac output. Data obtained from this monitoring device will also be available for use to the patients’ attending clinicians if they wish.

### Thromboelastography (ROTEM)

A 5 ml sample of blood will be withdrawn from existing vascular access devices and used to perform point-of-care analysis of coagulation status using the technique of thromboelastography (ROTEM). The following values will be recorded: EXTEM/FIBTEM CFT, A5, A10, MCF, LY 30.

### Biological sampling

Samples of blood and urine will be withdrawn from existing vascular access devices and stored for later assessment of the mechanisms of microcirculatory dysfunction.

### Physiological and pharmacological data

The following parameters will also be recorded at every time point where available:

- SBP, diastolic blood pressure and mean arterial blood pressure;
- Central venous pressure;
- Heart rate;
- Arterial Blood Gas Data (including lactate, base deficit, arterial oxygen saturation, arterial carbon dioxide saturation)
- Haemoglobin concentration;
- Central Venous Blood Gas Data (central venous oxygen saturation (ScvO_2_), central venous carbon dioxide saturation (ScvCO_2_));
- Plasma (free) haemoglobin;
- Administration of blood, blood products and bolus challenges of other intravenous fluids since the previous time point;
- Type of sedative drug infusions currently being administered;
- Type and dose of inotropic and vasopressor drugs currently being administered.

### Primary outcome

The primary outcome is the Sequential Organ Failure Assessment (SOFA) score at the D3 time point (72 h after admission to ICU), and will be recorded using information from the patients’ charts and laboratory results.

### Secondary outcomes

Secondary outcomes include SOFA scores and multiorgan dysfunction score on days 1, 7 and 28 of hospital admission (if the patient is still an inpatient), mortality at 28 days, length of stay (LOS) in hospital, LOS in ICU, number of days of mechanical ventilation, thromboelastography parameters, arterial and venous blood gas parameters, and blood product requirements.

### Analysis of IDF video sequences

Prior to analysis, the recorded video sequences will be edited into at least three (and no more than five) video clips. All clips will be rated for quality using a five-point scale.^17^ Substandard images will be rejected. All IDF images will be analysed using a dedicated software tool (Automated Vascular Analysis V.3.02, Microvision Medical, The Netherlands). This is a semiautomated process which produces a number of data points from the video sequence. Analysis of the video clips will produce the following data:

- Microvascular flow index;
- Total vessel density;
- Perfused vessel density;
- Proportion of perfused vessels
- Microcirculatory heterogeneity index.

This process is in accordance with recommendations made by an international consensus conference on assessing the microcirculation.^10^ Interobserver variation will be tested at regular intervals in order to minimise heterogeneity of data analysis between investigators. At the time of analysis, the investigators will be blinded with respect to the clinical time point of the study, the patients’ details, and the other outcome variables (SOFA score and haemodynamic data).

The following relationships will be examined:

- Between microcirculatory parameters during the postresuscitation period and the development of SOFA scores;
- Between microcirculatory parameters and systemic haemodynamic data (stroke volume, cardiac output, blood pressure);
- Between microcirculatory parameters and plasma lactate and ScvO_2_ over the resuscitation period;
- Between microcirculatory parameters and coagulation parameters as assessed by ROTEM.

### Capacity and consent

This study complies with the Declaration of Helsinki, and will be conducted in accordance with the principals of Good Clinical Research Practice (GCP). In order to assess the response of the microcirculation to haemorrhagic shock and resuscitation, intubated and ventilated patients will be recruited to the study at an early stage following their admission to hospital, when capacity for informed consent will be absent.

Lack of patient capacity will be addressed by referring to the Mental Health Capacity Act (2005). In this situation, the physician in charge of the care of the patient (nominated consultee) will be consulted in order to consent on behalf of the patient. These physicians will have received prior briefing on the study protocol, and will not be a study investigator. If possible consent will also be sought from a personal consultee (the next of kin, relative or friend of the patient). When the patient regains capacity, the study will be explained and the participant asked for their consent to use the data collected so far, as well as any further samples required by the protocol. If the participant declines consent and wishes to be removed from the study, their data will be erased and no further samples will be collected.

**Figure 2:**
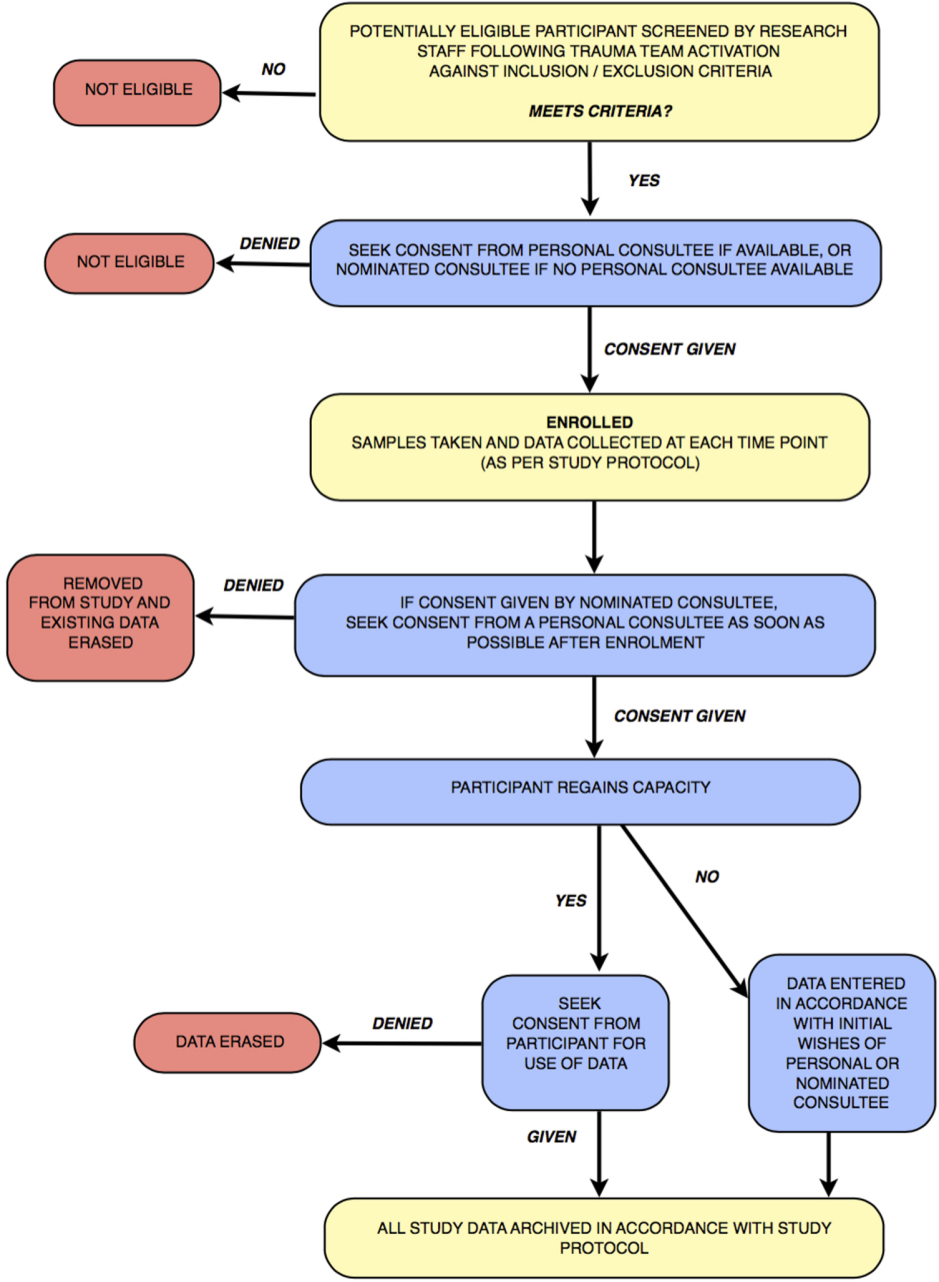
Process flow chart.

Patients who die or who do not regain capacity within 28 days of injury will have their data entered into the study in accordance with the wishes of their personal or nominated consultee. This process is summarised in figure 2.

### Confidentiality, data storage and security

All data relating to study participants will be treated in accordance with the Data Protection Act, 1998. All study data will be stored on a secure encrypted laptop used exclusively by the study investigators. Patients will be assigned an alphanumeric sequential study number that will be used to identify all clinical data. These numbers will be specific to each site. The patients’ demographic details will be linked to the study number on a separate screening database held on the secure computer at each site. Individual IDF image sequences will be assigned a random five-digit number. Identification of these video sequences will be achieved by cross-reference with an electronic database. On completion of the study, all participant identifying information and other study data will be securely archived in accordance with the policy of the sponsoring institution. All biological samples will be identifiable only by study number, and once analysis is complete the samples will be disposed off.

### Safety

The chief investigator has overall responsibility for the conduct of the study including responsibility for safety. Individual investigators will be responsible for reporting all serious adverse events (SAEs) and adverse events (AEs) to the chief investigator. There are no reported AEs associated with the use of IDF imaging in the published literature, and the risk of harm occurring to a study participant as a direct result of undergoing IDF imaging is close to zero. An example of an attributable AE would be a sublingual haematoma following IDF microscopy. SAEs will include death, any event which poses a threat to life, prolongs hospital stay or produces significant disability. Given the nature of the study population, it is anticipated that such features will be relatively common as part to the pathological process of trauma, but not as a result of participation in the study. The chief investigator will review all events to decide if there is any causal link. If this is the case action will be taken in accordance with the principals of ICH GCP.

### Statistical analysis

Normally distributed data will be presented as mean and SD, and non-normally distributed data will be presented as median and IQR. For comparison, patients will be dichotomised according to day 3 SOFA score into those with a score ≥6 versus those with SOFA score <6. The score of 6 has been chosen due to its prognostic relevance^18^ and previous utilisation as an end point for haemorrhagic trauma patients.^16^ Further analysis will be conducted according to the change in SOFA scores between days 1 and 3, so that patients are dichotomised into SOFA score ‘improvers’ and ‘non-improvers’. This approach has also been used as an end point for haemorrhagic trauma patients.^19^ Groups will be compared using a two-tailed t test for normally distributed data and the Mann-Whitney U test for non-normally distributed data. A p value of <0.05 will be considered statistically significant.

## Discussion

Bedside point-of-care technology that enables direct visualisation of the microcirculation offers an opportunity to gain insight into the real-time behaviour of the site of oxygen and nutrition exchange during shock. Although this has been primarily been a research tool in the past, advances in microcirculatory imaging may pave the way for a future clinical role.^20^ In particular because real-time assessment of images by clinicians may be as reliable as detailed offline computer analysis.^21^

Further research into bedside microcirculatory imaging, and the assessment of microcirculatory parameters as end points for resuscitation have been recommended by other investigators.^22^ The current study aims to address important questions with regard to the microcirculation during THS. Of particular interest is to investigate any immediate microcirculatory derangement on arrival in hospital following traumatic haemorrhagic injury, and whether there is a relationship between these changes and clinical outcomes. It is also not fully known whether there is a persistent microcirculatory derangement following initial resuscitation, and whether there is a relationship between this and subsequent organ dysfunction. Only one clinical study has reported such a finding,^16^ but this was a relatively small study, with high usage of vasopressors, which is a relatively uncommon practice in the UK. Further data are required in order to fully answer this research question. If there is a true lack of coherence between macro haemodynamic parameters (eg, cardiac output and blood pressure) when compared with microcirculatory parameters, then this may strengthen the argument to improve techniques and technology to enable bedside microcirculatory monitoring. The results of this study will be examined with these clinical questions in mind.

Trauma patients are heterogeneous, with multiple confounding variables that make interpretation of data more difficult than an animal model may offer. Nevertheless, a clinical study is both timely and necessary in order to start the process of translating what has been learnt from animal research into useful and clinically relevant knowledge. The Microshock study will be the first to enrol patients with THS as soon as they arrive in hospital. Measuring microcirculatory parameters this soon after injury offers an opportunity to gain a greater insight into the early processes and mechanisms of microcirculatory dysfunction in this context. If clinically relevant details are discovered, then this may enable future investigators to determine the priorities and processes that may be put in place to move the technology towards clinical utilisation.
